# Challenges for Private LTC Service Providers for Preventing Elder Abuse

**DOI:** 10.1093/geroni/igaa057.2378

**Published:** 2020-12-16

**Authors:** Naoki Ikeda, Noriko Tsukada

**Affiliations:** 1 Kamihonnmachi Law Office, Tennouji-Ku, Osaka, Japan; 2 Nihon University, Tokyo, Tokyo, Japan

## Abstract

his paper aims to identify factors that differentiate long-term care (LTC) service providers into the two categories: those who are successfully growing and those who are going out of business. During the past 5 years, about one out of 100 private LTC service providers has gone bankrupt in part due to issues of client abuse. This paper uses case studies to demonstrate differences between LTC service providers who have histories of elder abuse and those that do not. Business traits such as mission, client trust, and quality of LTC workers is considered along with implementation of abuse prevention practices including management approaches, oversight of workers, and atmosphere where LTC workers have their working conditions and concerns addressed, which in turn enhances workers satisfactions, accordingly, yielding better quality of care provisions.

